# Clinical Performance of Toris K Contact Lens in Patients with Moderate to Advanced Keratoconus: A Real Life Retrospective Analysis

**DOI:** 10.1155/2016/2358901

**Published:** 2016-04-06

**Authors:** Ihsan Yilmaz, Ferah Ozcelik, Berna Basarir, Gokhan Demir, Gonul Durusoy, Muhittin Taskapili

**Affiliations:** Beyoglu Eye Training and Research Hospital, 34420 Istanbul, Turkey

## Abstract

*Objectives.* To evaluate the visual performance of Toris K soft contact lenses in patients with moderate-to-advanced keratoconus and also to compare the results according to cone types, cone location, and severity of keratoconus.* Materials and Methods.* Sixty eyes of 40 participants were included in this retrospective study. Uncorrected visual acuity (UCVA), best-spectacle corrected visual acuity (BCVA), best-contact lens corrected visual acuity (BCLCVA), and comfort rating via visual analogue scales (VAS) were measured.* Results.* The mean age was 27.3 ± 8.6 years (range: 18 to 54). The mean logMAR UCVA, BCVA, and BCLCVA were 0.85 ± 0.38 (range: 0.30–1.30), 0.47 ± 0.27 (range: 0.10–1.30), and 0.16 ± 0.20 (range: 0–1.00). There were significant increases in visual acuities with contact lenses (*p* < .05). BCLCVA was significantly better in oval type than globus type (*p* = .022). UCVA and BCLCVA were significantly better in moderate keratoconus group (*p* = .015, *p* = .018). The mean line gain in Snellen was 3.6 ± 1.8 lines (range: 0–7 lines). The mean line gain was higher in central cone group than paracentral cone group and oval group than globus group (*p* = .014, *p* = .045). The mean VAS score was 8.14 ± 1.88 (range: 6–10).* Conclusions.* Toris K can improve visual acuity of patients with keratoconus. Toris K is successful even in the moderate and advanced form of the disease.

## 1. Introduction

Keratoconus is a progressive, noninflammatory ocular disorder characterized by steepening and distortion of the cornea, apical thinning, and central scarring [1]. It is generally bilateral and progresses asymmetrically in both eyes [2]. Progressive ectasia and thinning of the cornea result in irregular astigmatism and visual symptoms [3].

Spectacles are useful in optical management of the early stages of keratoconus when the astigmatism is mild [4]. However, for moderate-to-advanced keratoconus, spectacles are not very useful for improving vision [4, 5]. In this stage of the disease, when patients have serious irregular astigmatism, contact lenses become necessary [5]. Various options for contact lenses are available such as rigid gas permeable (RGP) lenses [6, 7], hybrid contact lenses [8, 9], piggyback lenses [10, 11], and scleral lenses [12, 13]. RGP lenses are traditionally the first choice and have been commonly used because of their success in improving visual acuity [7]. RPG lenses improve visual acuity by their refractive power and also by providing a regular corneal surface. However, prolonged use of RGP lenses may lead to lens intolerance and ocular discomfort in most patients with keratoconus [14]. Another important disadvantage of RPG lenses is the potential for damage to the cornea [15, 16]. Also sometimes, it is not possible to find the suitable RGP contact lens for each irregular corneal surface. In such cases, soft contact lenses should be considered. Toris K (SwissLens, Prilly, Switzerland) is an example of these kinds of lenses for keratoconus.

Soft contact lenses have their comfort advantages over RGPs but they do not provide a regular corneal surface as much as RGP lenses do. In this study, we aimed to evaluate the comfort and visual performance of Toris K soft contact lens in patients with moderate-to-advanced keratoconus. We also aimed to compare the visual performance of the contact lens in different cone shape (oval, nipplei globus), cone location (central, paracentral), and severity of the disease (moderate, advanced).

## 2. Methods

In this retrospective study, we reviewed the records of patients with keratoconus who were fitted Torsi K contact lenses between December 2013 and December 2015. Written informed consent was obtained from all patients before the contact lenses fit. The study adhered to the tenets of the Declaration of Helsinki and local ethic committee approval was obtained. To be included in the study, each patient was required to have all of the following criteria: age ≥ 18 years, a diagnosis of moderate-to-advanced keratoconus detected by thorough topographic evaluation in conjunction with the clinical examination, and mean corneal power >45 diopters (D).

Patients were not included in the study if they had a history of ocular surgery, history of ocular trauma, and any ocular disease (e.g., active ocular infection, clinically significant nuclear sclerosis/cataract, and retinal diseases) that might affect the results, and break-up time (BUT) under 10 seconds.

Data collected from the patients' records included age, gender, refractive errors, BUT, mean-steep-flat keratometric measurements and cone type-location from Sirius (Schwind eye-tech-solutions GmbH & Co. KG, Kleinostheim, Germany) scan, uncorrected visual acuity (UCVA), best spectacle corrected visual acuity (BCVA), best contact lens corrected visual acuity (BCLCVA), base curve (BC) prescribed contact lens diopter, and visual analogue scales (VAS).

All participants underwent a standardized ophthalmologic examination including refraction, visual acuity (Snellen from 6 meters via Topcon ACP-8 chart projector (Topcon Corporation, Tokyo, Japan) under photopic condition (85 cd/m^2^) luminance), slit-lamp biomicroscopy-fundoscopy, BUT, and corneal topography via Sirius.

After UCVA and BCVA were measured, contact lens was fitted as provided in its technical fitting guide and manufacturer's specifications were followed (Table 1). Toris K soft contact lens is a silicone hydrogel lens with front toric surface. It uses dynamic stabilization system with bumps at 0° and 180° engraved points. A dedicated set of lenses is needed for this purpose in different base curves and different peripheral radii with power. The lens was allowed to settle for approximately 5 min and then the movement, rotation, and centration were checked with a slit-lamp. After correct fit and patient comfort were achieved, residual refractive error was measured via retinoscopy. Then overrefraction was performed with correcting spectacle lenses and contact lenses were prescribed. BCLCVA and VAS score were measured a week later at the first visit of patients. VAS is previously used to rate comfort with contact lenses [14, 17]. VAS were administered by asking patients to record their subjective impressions of vision and comfort using a scale from 0 (lowest) to 10 (highest). The scale was horizontally oriented, measuring 10 cm, and the value for statistical analysis was measured with a rule at the point where the mark was inserted by the patient.

In this study, according to the topographic map, cone location was classified as central (if the highest power was located in central 2 mm) and paracentral (if the highest power was located out of central 2 mm). With Keratoconus classified based on the mean *K* reading on corneal topography, the patients were classified as mild in cases with *K* value less than 45 D, moderate in cases with *K* value between 45 and 52 D, and advanced in cases with *K* value more than 52 D [18, 19].

Primary outcome measures included UCVA, BCVA, BCLCVA, VAS, and results in subgroups according to cone location and severity of keratoconus.

### 2.1. Statistical Analysis

Visual acuity was converted to the logarithm of the minimum angle of resolution (logMAR) for statistical analysis. Categorical variables were presented as numbers and percentages, while numerical variables were expressed as the mean and standard deviation. The Kolmogorov-Smirnov test was applied to assess the normal distribution of data. The outcomes were compared using appropriate tests (paired-samples *t*-test, independent-samples *t*-test, and one-way ANOVA). The Statistical Package for the Social Sciences version 23 (SPSS, Chicago, IL, USA) was used for data analysis, for which values of *p* < .05 were considered to be statistically significant.

## 3. Results

### 3.1. Demographic Characteristics

The study sample consisted of 60 eyes of 40 participants (17 females and 23 males), all of whom were Caucasian. The mean age was 27.3 ± 8.6 years (range: 18 to 54).  Table 2 shows participants' demographic characteristics.

HydroCone K34 lenses were used for all participants. The mean base curve (BC) was 7.75 ± 0.15 (range: 7.40 to 8.20). The mean spherical and cylindrical power (D) of prescribed contact lens were −2.89 ± 2.43 (range: −10 to 1) and −2.02 ± 1.08 (range: −4.25 to 0).

### 3.2. Visual Acuity

Table 3 shows the visual acuities. There was a significant difference between BCVA and BCLCVA (*p* = .000).

When we divided the patients into subgroups according to cone type, there was no significant difference in UCVA and BCVA between groups (*p* = .091, *p* = .817, one-way ANOVA) but there was a significant difference in BCLCVA between groups (*p* = .029, one-way ANOVA). Post hoc Tukey test showed that BCLCVA was significantly better in oval type than globus type (*p* = .022).

When we divided the patients into subgroups according to cone location, there was no significant difference in UCVA, BCVA, and BCLCVA between groups (*p* = .610; *p* = .630; *p* = .468 (independent-samples *t*-test)).

When we divided the patients into subgroups according to the severity of keratoconus, UCVA and BCLCVA were significantly better in moderate keratoconus group (*p* = .015, *p* = .018, independent-samples *t*-test). There was no difference in BCVA between groups (*p* = .085).

**Figure 1:**
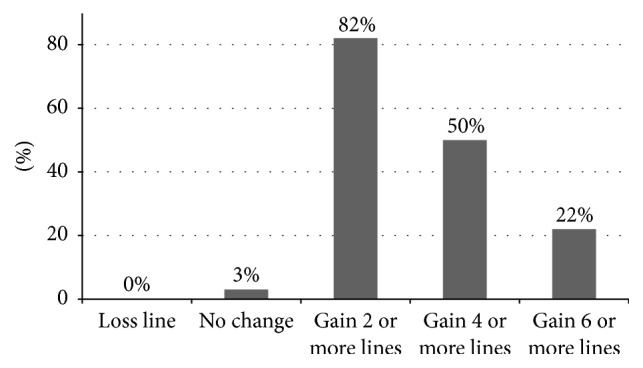
Line gains in Snellen with contact lens (difference between BCLCVA and BCVA).

Figure 1 shows line gains in Snellen. The mean line gain in Snellen between BCVA and BCLCVA was 3.6 ± 1.8 lines (range: 0–7 lines). 45 patients (75%) gained 3 or more lines with contact lens correction against spectacle correction. Two patients (3.33%) gained no lines but gained only 2 letters. However, those patients expressed that their subjective vision was better with contact lens. There was no significant difference in the mean line gain between moderate keratoconus group and advanced keratoconus group (*p* = .104, independent-samples *t*-test). The mean line gain was higher in central cone group than paracentral cone group (*p* = .014, independent samples *t*-test). There was significant difference in the mean line gain between oval-nipple-globus groups (*p* = .045, one-way ANOVA). The post hoc Tukey test showed that the difference was between oval and globus type and the mean gain line was significantly higher in oval group (*p* = .045).

The mean VAS score was 8.14 ± 1.88 (range: 6–10).

## 4. Discussion

Contact lenses have an important role in the management of visual symptoms of patients with keratoconus. The purpose of fitting contact lenses in such patients is to improve visual acuity with comfort [4]. It is well known that RPG contact lenses produce good visual acuity results and improve patients' quality of life [7]. Yazar et al. showed that RPG contact lenses may cause some serious complications such as corneal erosion, hidrops, allergic conjunctivitis, and dry eye [20]. Another downside of RPG lenses is that many patients cannot tolerate them because of comfort issues [8]. Soft contact lenses are known for comfort and they may be a good alternative to RGP lenses in patients who experienced intolerance to RGP lenses [21].

Gumus and Kahraman reported results of Toris K contact lens in keratoconus [22]. In their study, comfort score was classified as good/excellent in 46 eyes (92% of participants) and moderate in only 4 eyes (8% of participants) [22]. Similarly, comfort scores were high in our study. The high VAS score provides strong evidence that patients who cannot tolerate RGP lenses due to discomfort may find Toris K contact lens an acceptable option.

In Gumus and Kahraman study, the mean increase in visual acuity was reported 4.5 lines (range: 1 to 9 lines). 92% of their participants classified their visual acuity as good/excellent in daytime, and 76% were classified as good/excellent in nighttime [22]. In the present study, the mean visual acuity was significantly better with the contact lens than with spectacles and improved to 0.16 ± 0.20 logMAR from 0.47 ± 0.27 logMAR. The mean line gain was over 3 lines. Good visual results show to us that Toris K is effective for treatment of visual symptom of moderate-to-advanced keratoconus.

**Figure 2:**
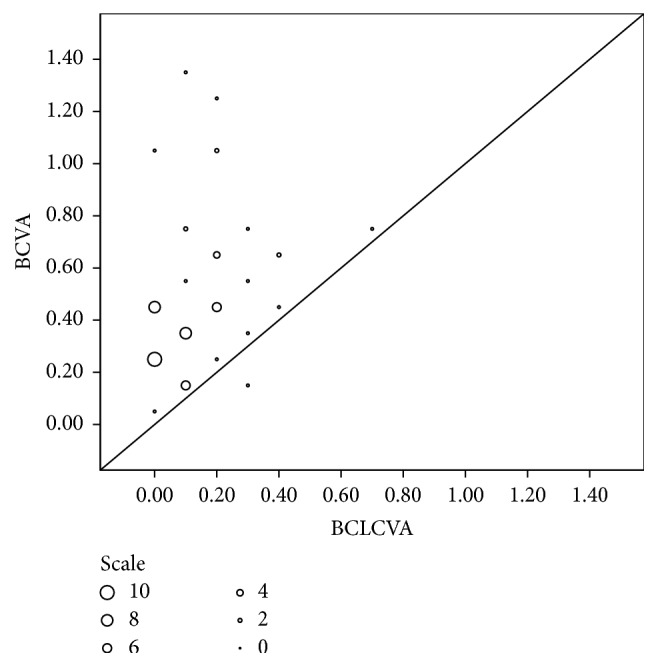
BCLCVA versus BCVA (logMAR).

This study is the first study in literature that compares the visual results of Toris K contact lens fitting in keratoconus subtypes (cone type, cone location, and severity of keratoconus). Romero-Jiménez et al. compared the optimal lens fit rates between nipple and oval cones in the literature [23]. We added visual results comparisons in keratoconus subtypes to the literature. Results show us that BCLCVA and the mean line gain were better in oval cones than globus cones (Figure 2). We still got improvements with contact lens in globus group. However, we believe that visual management of globus type cones is harder than other types with soft contact lenses and another contact lens type or surgical options may be necessary.

The mean visual acuity with contact lens was better in moderate keratoconus group than advanced group. It is proved that contact lens vision correction is more successful at the early stages of the disease.

Nejabat et al. showed that the cone location has no effect on the RGP corrected visual acuities in patients with keratoconus [19]. Similarly, this study showed that the cone location (central or paracentral) has no effect on the Toris K soft contact lens corrected visual acuities. However, the mean gain line was higher in central cone group.

This study has limitations. Retrospective nature of the study is a limitation. Also this study does not show the lens behaviors, complications, in the long term. It is well-known that soft contact lenses are thick and cause some complications, due to hypoxia, such as corneal swelling, contact lens induced papillary conjunctivitis, and superior punctuate keratitis [24, 25].

The strongest aspect of the study is that being first report in the literature that examines the visual results of Toris K contact lens in different subtypes (cone type, cone location, and severity of keratoconus) of keratoconus. Finally, reduced sample size in some subgroup comparisons may count as a limitation.

In sum, Toris K contact lenses may improve visual acuity with comfort in patients with keratoconus. Toris K is successful even in the moderate and advanced form of the disease. Further studies with longer follow-up period that compared the different soft contact lenses are needed.

## Figures and Tables

**Table 1:** The fitting set parameters and the fitting assessment procedure of Toris K.

Technical data	Values
Total diameter	13.70 mm (HydroCone K12)
	14.00 mm (HydroCone K34)

Base curve	7.20 to 8.40 D

Sphere	−40.0 to +40.0 D

Cylinder	−0.01 to −8.00 D

Axis	0–180°

Center thickness	Standard K12 = 0.42 mm, K34 = 0.52 mm
	Range of thickness: 0.3520.59 mm

*The Fitting Assessment Procedure*	
First contact lens choice	
It is suggested working with trial lenses with cylindrical power −0.01 D	
Keratoconus classification	
First apply topographical indications or follow the rules:	
Vcc > 0.6 and/or keratometry > 6.8: grade 1 or 2 (choose HydroCone K12)	
Vcc < 0.6 and/or keratometry < 6.8: grade 3 or 4 (choose HydroCone K34)	
Diameter and base curve selection	
Add 0.8 diopters to the average *K* value and then select a trial lens	
HydroCone K12/total diameter = 14.00 mm	
HydroCone K34/total diameter = 13.70 mm	
Further steps	
The first lens helps to validate base curve and total diameter	
The patient should wait for 30 min	
Dynamic stabilization marks should be evaluated to measure the stabilization axis	
Push-up test should be done	
The fitting curve should demonstrate typical fitting of characteristics of a standard soft lens fit	
If the fitting curve is too flat, there will be excessive movement and/or edge lift and then switch to a steeper trial lens	
Little or no movement and/or edge impingement would indicate the fitting curve is too steep and then switch to a flatter trial lens	
Overrefraction should be done	
Prescription should include both spherical and cylindrical errors with its axis	
In case of glare and/or halo, you can request to enlarge the optical zone	

D: diopter and Vcc: best spectacle corrected visual acuity.

**Table 2:** Demographic information of the population enrolled in the study.

Parameter	Values
Number of patients	*n* = 40
Females	17 (42.5%)
Males	23 (57.5%)
Age	
(mean ± sd)	27.3 ± 8.6
(min./max.)	18/54
Number of eyes	*n* = 60
Bilateral	20 patients
Unilateral	20 patients
Refractive error (D)	*n* = 46
Spherical	
(mean ± sd)	−3.31 ± 3.62
(min./max.)	−15/2.75
Cylindrical	
(mean ± sd)	−3.94 ± 1.72
(min./max.)	−8/0
Keratometry (D)	*n* = 60
Flat	
(mean ± sd)	47.45 ± 3.56
(min./max.)	42.72/60.27
Steep	
(mean ± sd)	50.85 ± 3.89
(min./max.)	46.10/63.82
SimK	
(mean ± sd)	49.10 ± 3.66
(min./max.)	45.02/61.72

D: diopter and sd: standard deviation.

**Table 3:** Visual acuity (logMAR, mean ± sd, min./max.).

	UCVA	BCVA	BCLCVA	Mean gain line
All eyes	0.85 ± 0.38	0.47 ± 0.27	0.16 ± 0.20	3.6 ± 1.8
	0.30/1.30	0.10/1.30	0/1.00	0/7
Cone types				
Oval (*n* = 18, 30%)	0.79 ± 0.30	0.43 ± 0.23	0.08 ± 0.09	4.0 ± 1.7
	0.30/1.30	0.10/1.00	0.00/0.20	1/7
Nipple (*n* = 31, 51.67%)	0.81 ± 0.40	0.48 ± 0.31	0.14 ± 0.19	3.7 ± 1.8
	0.30/1.30	0.10/1.30	0.00/1.00	0/6
Globus (*n* = 11, 18.33%)	1.08 ± 0.36	0.49 ± 0.20	0.25 ± 0.20	2.4 ± 1.6
	0.30/1.30	0.20/0.70	0.00/0.70	0/5
Cone location				
Central (*n* = 48, 80%)	0.85 ± 0.38	0.47 ± 0.28	0.13 ± 0.18	3.8 ± 1.8
	0.30/1.30	0.10/1.30	0.00/1.00	0/7
Paracentral (*n* = 12, 20%)	0.91 ± 0.38	0.43 ± 0.24	0.17 ± 0.14	2.4 ± 1.2
	0.30/1.30	0.10/0.70	0.00/0.40	1/4
Severity of keratoconus				
Moderate (*n* = 50, 83.3%)	0.81 ± 0.36	0.44 ± 0.25	0.12 ± 0.16	3.7 ± 1.8
	0.30/1.30	0.10/1.30	0.00/1.00	0/7
Advanced (*n* = 10, 16.7%)	1.12 ± 0.38	0.60 ± 0.32	0.26 ± 0.20	2.7 ± 1.8
	0.30/1.30	0.20/1.30	0.00/0.70	0/6

UCVA: uncorrected visual acuity, BCVA: best-corrected visual acuity, BCLCVA: best contact lens corrected visual acuity, sd: standard deviation, and D: diopter.
